# The convergence of health system, climate, and social pathways in Malawi’s 2022–2024 cholera outbreak – A qualitative assessment

**DOI:** 10.1371/journal.pntd.0014529

**Published:** 2026-07-10

**Authors:** Shahar Livne, Matamando Mwendera, Stellar Chibvunde, Moses Aron, Nadav Davidovitch, Fabien Munyaneza, Anat Rosenthal

**Affiliations:** 1 Department of Health Policy and Management, School of Public Health, Faculty of Health Sciences, Ben-Gurion University of the Negev, Beer Sheva, Israel; 2 Partners in Health/Abwenzi Pa Za Umoyo, Neno, Malawi; 3 Faculty of Health Sciences and Science and Technology Program, Bar Ilan University, Ramat Gan, Israel; 4 Swiss Tropical and Public Health Institute, Allschwil, Switzerland; Kwame Nkrumah University of Science and Technology, GHANA

## Abstract

Climate-related disasters increase cholera risks, yet their contribution to outbreaks is often explained through microbiological, epidemiological or ecological lenses, limiting our understanding of system convergence. Examining the 2022–2024 cholera outbreak in Malawi, we explore how healthcare providers in two rural districts perceived the interconnected pathways that transformed a seasonal cholera outbreak into a two-year epidemic. Drawing from field visits in Neno and Chikwawa Districts of Malawi, we conducted 24 in-depth semi-structured interviews with first responders. We identified three converging pathways: tropical cyclones that disrupted traditional disease patterns and recovery capacity; health system vulnerabilities characterized by growing unpredictability and infrastructure destruction; and social and economic determinants of health including mass displacement, inadequate water, sanitation, and hygiene infrastructure, and cross-border transmission. The convergence of these pathways amplified acute climate impacts and chronic structural vulnerabilities. These findings highlight the need for integrated system approaches that address pathway interconnections rather than isolated interventions in cholera containment, essential for meeting 2030 elimination goals.

## Author summary

Climatic disasters frequently trigger cholera outbreaks in low-income settings, yet the structural and social pathways that sustain these outbreaks into large-scale epidemics are often overlooked from a system-thinking perspective. In this study, we aimed to understand how first responders in rural Malawi perceived the drivers that transformed a seasonal outbreak into the deadliest cholera outbreak in the country’s history. Through field visits, observations, and 24 in-depth interviews, we identified three converging pathways. First, cyclones disrupted traditional disease patterns and altered the system’s anticipation capacity. Second, these climate impacts exposed and created new health system vulnerabilities. Third, social and economic factors converged, including mass displacement, limited access to water and sanitation, and cross-border transmission. These overlapping challenges amplified existing structural weaknesses, making containment difficult and the goal of eliminating the disease by 2030 even more so. Our results suggest that prevention and control strategies should address these pathways and their convergence in both planning and practice.

## Introduction

Cholera is a waterborne, bacterial, infectious disease responsible for 2.86 million cases a year and an estimated 100,000 deaths worldwide. As a disease that fundamentally reflects global inequality, cholera disproportionately impacts Sub-Saharan Africa (SSA), with over 60% of cases occurring in this region [1]. With outbreaks increasing in recent years, especially in areas with limited access to clean water, and slow progress in water, sanitation, and hygiene (WASH) infrastructures, the road to eliminate cholera by 2030 appears increasingly distant [2–5].

In addition, the climate crisis serves as a critical amplifier of cholera outbreak risks through multiple interconnected pathways, as rising water temperatures [6,7], and extreme weather events correlate with increased cholera transmission through both droughts and floods [8–10]. However, research from SSA shows a variation in outbreak patterns, indicating that cholera dynamics are complicated and extend far beyond climate change and inadequate WASH infrastructure alone [5,10,11].

This complexity necessitates better integration across disciplinary boundaries and sectors [8,12]. Nevertheless, cholera continues to be examined through mainly disciplinary silos, with biomedical or epidemiological approaches remaining the leading ones. As Bedford et al. [13] noted: “Despite great technological progress and expansion of the field, the theories and practices of infectious disease epidemiology are struggling to keep pace with the transitional nature of epidemics in the twenty-first century and the breadth of skills needed to respond to them” (p.130). While established frameworks such as One Health and various system risk models have begun to bridge these gaps, they often rely on high-level policy analysis or ecological data. These challenges necessitate the integration of multiple disciplines and adoption of qualitative methods that can illuminate how different systems interact during infectious disease outbreaks and how these interactions affect risk and care [13–16]. In this paper, we therefore move beyond standalone biomedical, epidemiological or ecological models to offer an integrated system approach [14,17,18] that can better capture the multifaceted nature of cholera transmission in disaster contexts.

**Fig 1:**
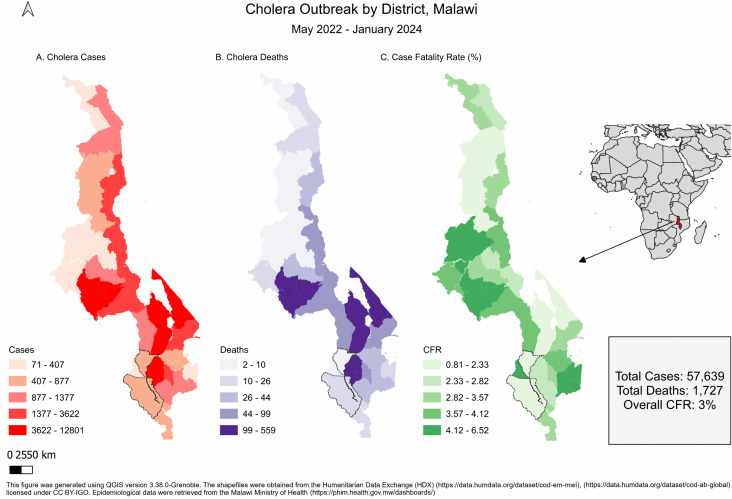
Cholera outbreak by districts, Malawi. Sources: The figure was generated using QGIS version 3.38.0-Grenoble. The shapefiles were obtained from the Humanitarian Data Exchange (HDX) (https://data.humdata.org/dataset/cod-em-mwi), (https://data.humdata.org/dataset/cod-ab-global) licensed under CC BY-IGO. Epidemiological data were retrieved from the Malawi Ministry of Health (https://phim.health.gov.mw/dashboards/).

From this perspective, the cholera outbreak of 2022–2024 in Malawi provides a compelling case study of a climate-induced outbreak, as it was exacerbated by the conditions created by three cascading cyclones: Cyclone Ana in January 2022, Cyclone Gombe in March 2022, and Cyclone Freddy in 2023, two of which (Ana and Freddy) were the most severe Malawi has ever experienced [19]. The 2022–2024 cholera outbreak is considered the deadliest in the country’s history, with cases reported across all 29 districts, and with a total of 57,639 cases and 1,727 deaths (Fig 1) (Malawi Ministry of Health, 2024). The outbreak was further intensified by the introduction of a new strain of Vibrio cholerae, which is more contagious than the seasonal strains generally contended with by the health system [20]. Combined with environmental devastation and millions of cyclone-affected populations, these factors created fertile ground for the widespread transmission of the disease [14,21].

The Malawian health system faced immense challenges in delivering services amid these interconnected crises: responding to cyclone aftermaths, the cholera outbreak, and maintaining routine healthcare in an already resource-constrained setting [22]. The situation was particularly severe in the Southern Region of the country, where the cyclones caused the most damage and where poverty levels are the highest, and WASH infrastructures were already inadequate [23,24]. In this rural region, 65% of the population relies on boreholes for water usage, and only 3% have flush toilets at home. In Chikwawa District, the hardest hit district, 10.7% of households lacked any toilet facility before the cyclones in 2018 [25], conditions that further deteriorated as a result of the cyclones’ impacts [23,24]. However, these statistics provide only a partial view of the factors that contributed to the outbreak. They do not explain how social and economic determinants of health interacted with climate-related disaster pathways and health system realities to create conditions for disease transmission.

These realities include health systems that are already under-resourced and contending with competing priorities, staff shortages, and supply constraints alongside epidemic control [26]. Studies examining the role of fragile or resource-limited health systems during cholera outbreaks point to lack of preparedness, insufficient collaboration with communities and stakeholders, difficulties in case management, and challenges in epidemic control amidst conditions such as internally displaced person (IDP) camps with inadequate sanitation [27–32]. Although these studies have provided valuable insights, they tend to examine health system strengthening in isolation rather than exploring how health, environmental, and social systems interact.

Context-specific analyses of such systemic interactions in Malawi remain scarce. A recent and significant exception is the work by Kita et al. which examines systemic risks during the COVID-19 pandemic in Malawi to highlight how pre-existing vulnerabilities were amplified by concurrent hazards [33]. However, it focuses primarily on high-level policy analysis. Healthcare provider perspectives on these pathway interconnections remain largely unexplored, as does research on how health systems learn and adapt between recurrent climate-related outbreaks [34,35].

We aim to address these gaps by examining healthcare provider perspectives on pathway interconnections during Malawi’s 2022–2024 cholera outbreak. We argue that the interaction of social vulnerabilities with the mass destruction caused by extreme weather events in an already limited healthcare system, creates feedback loops that amplify transmission. The complexity of these feedback loops necessitates shifting from linear to complex-adaptive-systems thinking for cholera prevention, focusing not only on disease control but also on other essential services and needs. Healthcare providers, who witness firsthand how these pathways converge during crises, offer critical insights for developing more effective, integrated prevention and response strategies essential for achieving cholera elimination goals.

## Methods

### Ethics statement

The study was approved by the Ethics Committee of the Malawian National Health Sciences Research Committee (NHSRC Protocol #23/12/4270), the Neno and Chikwawa Districts Health Research Committee, and the Ben-Gurion University of the Negev Human Subjects Research Committee (#275-1). All participants provided written consent to be interviewed and audio-recorded, after receiving an explanation of their rights and assurance that they could withdraw from the study at any time. We assigned unique numbers to audios and transcripts to ensure de-identification and securely stored them on password-protected computers only accessible to the research team.

### Study setting

The study was conducted in Neno and Chikwawa Districts, both rural and hard-to-reach areas in Southern Malawi. Neno District is a mountainous district with a population of about 166,000 residents, and Chikwawa District lies in the lower Shire River and has a population of 566,000 who primarily work in small-scale rain-fed agriculture [36,37]. These districts were purposefully selected to capture the diversity of rural challenges in controlling of the outbreak. While both are categorized as rural and “vulnerable”, they represent different topographical and hydrological risks. Notably, the riverine flooding in Chikwawa led to the highest concentration of IDP camps in the region following Cyclones Ana and Freddy [19].

Although very different in size, both districts have a very limited network of tarmac roads, and many areas become inaccessible during the rainy season. Both districts have experienced recurring cycles of droughts, floods, and tropical storms over the past decades [38]. These events have resulted in extensive damage to critical infrastructure, including road networks and healthcare facilities, housing, agriculture, and the local economy [23]. The resultant limited accessibility has exacerbated the vulnerability of local communities and facilities (such as hospitals and clinics) during disasters and has also slowed down disaster response [39].

### Study design

The results reported in this article are based on data that were collected using qualitative research methods in August-September 2024, i.e., 2.5 years after the onset of the cholera outbreak that followed cyclone Ana. The broader study included the collection of mix-methods data in communities and healthcare facilities. The present article is based on a Qualitative Descriptive study [40] that included 24 in-depth interviews with first responders in Neno and Chikwawa Districts. This qualitative approach gave us insights into how first responders in the two districts understood the post-cyclone cholera outbreaks, the pathway leading to them, and what they perceived to be needed in order to cope, recover, and prepare for future events.

We supplemented interview data with participant observations and site visits across multiple sites in both districts at different and varied geographic settings and levels of resource access in order to capture the diversity of factors influencing cholera outbreaks and responses as they appeared in the interviews.

### Study population

The participants recruited for in-depth interviews included 12 first responders from each district (N = 24). Participants ranged in specialities and included clinicians, environmental health specialists, health surveillance assistants, and persons in management positions (see Table 1). The purposeful sample of participants was used to address the variety of perspectives and experiences within this heterogeneous group of first responders. While the broader response involved various actors, including of communities and civil servants from other sectors, this study intentionally prioritized health workers as they serve as the primary link between national policy and community-level implementation. A few participants had pre-existing working relationships with the study team, particularly those in higher positions.

**Table 1:** Participant demographics by district.

Variable	Neno District	Chikwawa District	Total
**N**	**12**	**12**	**24**
**Gender**			
Male (%)	9 (75)	9 (75)	18 (75)
Female (%)	3 (25)	3 (25)	6 (25)
**Professional Role**			
Clinical (%)	7 (58.3)	6 (50)	13 (54.2)
Environmental (%)*	1 (8.3)	3 (25)	4 (16.7)
Management (%)*	6 (50)	5 (41.7)	11 (45.8)
Other	2 (16.7)	0	2 (8.3)
**Education Level**			
Secondary	0	1 (8.3)	1 (4.2)
Diploma (including clinical officer)	0	5 (41.7)	5 (20.8)
Higher Education	12 (100)	6 (50)	18 (75)

*Some participants may have had multiple roles, resulting in totals that may exceed 100%.

### Data collection

The in-depth interviews were conducted by SL and AR, a PhD student and her advisor, both experienced female qualitative researchers. They used semi-structured interview guides with open-ended questions about the cyclones, the subsequent and ongoing cholera outbreaks, and the participants’ roles as first responders. The interviews were conducted in English using interview guides that were adapted to the local terminology. The interview guide was tested through pilot interviews within all team members and with various stakeholders. The recruitment process emphasized diversity of specialties and roles as first responders, gender, age, and experiences. Interviews were conducted in private rooms at the participants’ workplaces and lasted between 45 and 90 minutes.

In addition to the interviews, SL, AR, SM, MM, and MBA conducted field visits at local hospitals and clinics as well as at the cholera treatment center sites, locations of past outbreaks, and the local offices of first responders. Each visit lasted between 30–60 minutes, and included assessments of infrastructure and access to services.

### Data analysis

After reaching data saturation, we transcribed the interviews and prepared them for coding and analysis using Dedoose qualitative data analysis software. The systematic coding process included multiple phases. In the first phase, the team developed a preliminary codebook based on our research questions, the existing literature and immersion in the collected data (interviews and field notes). In the second phase, a subset of transcripts was coded by multiple team members (AR, SL, MM) using the preliminary codebook to establish coding consistency. The research team then met to discuss coding discrepancies, identify language gaps, refine definitions, and revise the codebook accordingly. Once the codebook was updated, the entire data set of interviews was coded by multiple team members using the new revised codebook. The collected data were thematically analyzed, organizing the written material in codes and themes based on the ideas extracted from the text [41].

Themes were created and categorized based on the frequency, extensiveness, and intensity of the quotations and their relation to each other, to allow cross-referencing and triangulation of findings. The themes developed during the analysis phase were used to map the pathways of risk and vulnerabilities as presented in this article.

### Reflexivity statement

The current study was conducted in collaboration with Partners in Health (PIH) and its local branch in Malawi, Abwenzi Pa Za Umoyo (APZU), an international social justice organization that has operated in Neno, Malawi since 2007.

The research team consisted of seven professionals from diverse personal and academic backgrounds, holding Malawian, Rwandan, and Israeli nationalities. Our collective expertise spanned clinical medicine, public health, medical anthropology, epidemiology, and critical disaster studies, coupled with extensive experience of working with the Malawian health system. The research Co-Principal Investigators were AR and FB, and the first author, SL, is a PhD student.

This team diversity was essential for triangulating perspectives, particularly in understanding the interconnectedness of pathways and gaining insider insights into the health system during disasters. This was further reinforced by the experience the Malawian-based team members acquired as responders to climate and health emergencies in Malawi prior to the research as part of their work with PIH/APZU. The collaboration with PIH/APZU facilitated access to the study sites, the development of relevant and locally grounded research questions and tools, and the triangulation of themes. However, this collaboration may have affected participants’ responses, as they may have identified the research team with the organization. Therefore, we proactively employed several strategies to address these potential biases. Firstly, interviews were conducted by SL and AR, two foreign researchers who are not part of PIH/APZU. Second, we interviewed participants from a variety of healthcare facilities. Third, we conducted participant observations to contextualize and supplement interviews’ findings, and forth, throughout the design, collection, and analysis, we engaged in continuous team discussions to identify and mitigate potential biases, thereby enhancing the trustworthiness of the qualitative findings.

## Results

**Fig 2:**
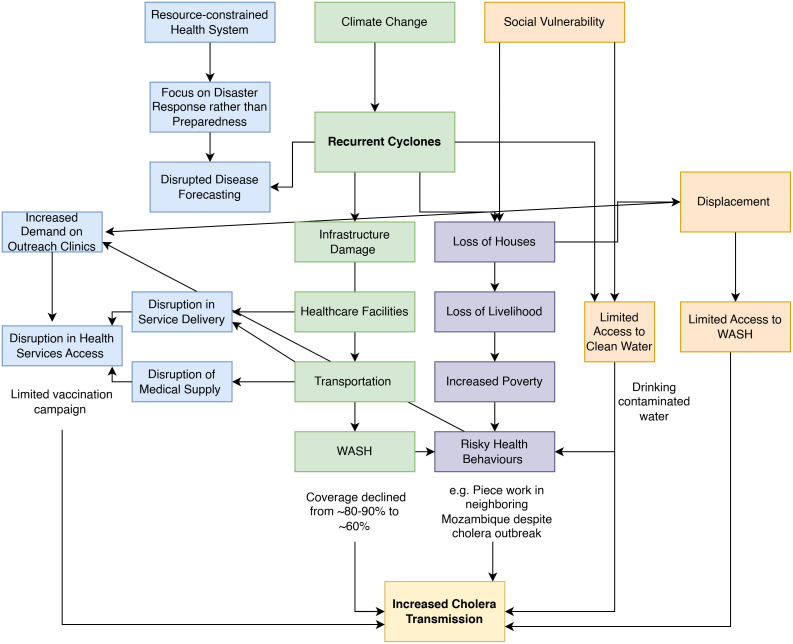
The convergence of health system, climatic, and social pathways in Malawi’s 2022-2024 cholera outbreak crisis.

Through our analysis of the interviews and field observations, we identified three distinct pathways that healthcare providers perceived as central to the prolonged and difficult-to-control cholera outbreak of 2022–2024 (Fig 2): a health system already stressed by multiple priorities and limited resources and staff; climatic variability and extreme weather events that affect water access, housing, and agricultural production; and poverty that constrains access to improved WASH infrastructure. Respondents identified complex interactions and feedback loops between these pathways, which created the conditions for the most devastating cholera outbreak in the country’s recorded history.

### The climate pathway

Healthcare providers identified a fundamental shift in both temporal and spatial changes of extreme weather events. A hospital attendant from Chikwawa, a long-time community hospital worker, explained the temporal changes he witnessed: *“*Usually, the rainy season was just normal. People planted and then they harvested their crops without much problems. But this time around, [from] 2014, we started experiencing these climate changes.” (140824SNL1)

Although floods and droughts have historically been recurrent hazards in Malawi due to El Niño and La Niña cycles [42,43], the last two decades have witnessed unprecedented increases in the frequency and intensity of extreme weather events [23]. From 2014 to 2023, more than twenty-five disasters associated with severe rainfall events occurred in Malawi, presenting an increasing trend in the number of people affected and in damage to critical infrastructure [23].

Healthcare providers identified different timepoints to mark this transition. Some, such as the hospital attendant, identified the floods of 2014 or 2015–2016 as the starting point; others pointed to Cyclone Idai in 2019 as when climatic disasters began to systematically affect their communities and facilities. The severity of these events is demonstrated by their cumulative effects. Participants pointed to the signs of damage that remain visible in clinics years after the floods, with electric sockets that remain nonfunctional below the water stains on the walls, or maternity wards that no longer operate.

Beyond temporal changes, healthcare providers also identified major changes in the geographical spread of extreme weather impacts. Areas previously not affected, such as those not situated in proximity to water basins or rivers that constitute traditional “flood-prone areas,” are now becoming more vulnerable to these hazards. A nurse from Neno described these spatial transformations:

It happens [climate-related disasters], and it doesn’t happen everywhere. It happens in the southern part of Malawi, which is in the Lower Shire basin, which is flood-prone as described by many. So we thought it was going to be the same [as in the past]. But then I think what made us realize that this was different was when we started seeing that actually the places which had never experienced such things were also impacted. And Neno was one of them. (200824ANZ1)

These temporal and spatial changes in extreme weather events disrupt weather predictions and preparation capacity. A nurse who has been working in Chikwawa District for over a decade described this challenge: “There is no choice of a place. It [an extreme weather event] can happen at any place, at any time. Whether you are expecting it or not expecting it. Because now we cannot predict it very well anymore.” (220824SCB2)

The shortened periods between successive disasters creates a critical constraint on system recovery and strengthening. As many of the healthcare providers emphasized, the frequency of events disrupts recovery processes, leaving no window of opportunity to improve preparedness and fully rehabilitate damaged infrastructure. The same nurse from Neno explained: “You don’t have enough time to do that [improve preparedness] when you [have to just] keep responding. So, if you move from one [disaster] to the next, there is no time in between for you to sit down and reflect” (200824ANZ1). A manager from a humanitarian organization captured the broader implications of this continuous disaster cycle:

I would say it’s catastrophic, because it has been continuous maybe for the past 10 years. We’re not looking only at the disasters themselves, because most of the times the disasters also come with other, different problems. You look at being affected consistently for 10 years; we’re looking at economic issues. We’re already in an economic crisis. (270824SCB1)

As this manager indicated, the destruction from the cyclones persists beyond the immediate effects, compounding impacts on food security and agricultural production, with cascading effects on human health. Additionally, given the country’s limited resources, the recovery process for housing, infrastructure, and healthcare facilities can last for years, worsening with every new cyclone that hits the region [23,24]. These interconnected impacts create direct and indirect pathways to worsening health conditions and infectious disease outbreaks. One particularly significant pathway involves the systematic decline in WASH infrastructure coverage, as an Integrated Disease Surveillance and Response coordinator from Neno described the cumulative effects of Cyclones Ana and Freddy:

As for me, from the experience that I have, I know that we can expect to see more cholera cases this year up to next year. Our latrine coverage currently is at 60% from somewhere around 73%. And we should expect more cases to do with gastroenteritis, be it cholera or any other diarrheal disease. And also, we should anticipate floods and other harsh weather conditions. (190824ANB1)

The impacts of intensifying extreme weather events extend far beyond the immediate period of destruction. Long-term effects persist due to limited national and district capacity to rehabilitate damaged infrastructure, compounded by increasingly shortened periods between climatic events that prevent adequate recovery and preparedness efforts.

### The health system pathway

In the context of the health system pathway, we identified three trends in the cholera outbreak: growing unpredictability, the role of extreme weather events as disease catalysts, and service disruption caused by infrastructure damage.

Study participants expressed deep concerns regarding their inability to plan health policy under rapidly changing, unfamiliar conditions. Relying on past weather and disease patterns to predict needs and allocate resources was no longer a safe course of action.

Participants described unseasonal weather patterns as factors shaping both the response and preparedness of communities, as well as the health system’s ability to prepare and respond to outbreaks. Addressing the unseasonality of climate patterns and their impact on communities, one participant explained:

We expect cholera when the rains are about to start. But this time, the cholera came in when it was not the season. It was out of season, [and] hit almost every district badly. And we even had deaths registered from cholera. It was very bad. (160924SNB2)

The overwhelming demands put on the already struggling health system resulted in a spiral of emergencies, as one participant explained:

To start with, in the past five years I think we have been hit by Cyclone Idai, Cyclone Ana, Cyclone Gombe, Cyclone Freddy, and the Dry Spell. But apart from that, in between we were also affected by other pandemics, including COVID-19, because it affected lives in the district and it was a global challenge, of course. And not only that. We have also been hit by cholera outbreaks as a result of the effects of climate change. We’ve had cholera, typhoid, and other water-related diseases. (190824SNB3)

The need to navigate changing weather patterns and new emergencies crystallized into a strong sense of unpredictability and the inability to plan ahead or focus disaster response efforts on preparedness and risk reduction. Following Cyclones Ana and Freddy, healthcare providers reported an increased prevalence of communicable diseases, due to the destruction of WASH infrastructure, overcrowded IDP camps, and contaminated floodwater.

Beyond the rise in communicable diseases in the post-disaster era, healthcare providers also raised concern about another trend of changing cholera patterns, from a seasonal endemic disease to a year-round disease. These changes have been attributed to social determinants of health, such as increased levels of poverty that are exacerbated by extreme weather and limited support:

Cholera, measles – we’ve been seeing a lot of outbreaks of conditions that were once controlled, but because of the natural disasters that we’ve been experiencing and the lower levels of livelihoods from communities and increasing levels of poverty, some of the conditions that were once suppressed are actually resurfacing. So, we are seeing things like measles outbreaks, cholera, which used to be confined to [the] rainy season, but this time around, actually even right now, there are cases that have been reported in certain parts of the country, which is quite unusual during the dry summer season. (190924SNB1)

These perceived changing and evolving disease patterns represent a shift in the epidemiological reality in Southern Malawi, challenging existing disease control strategies and surveillance systems. Another challenge is the increase in extreme weather events. Cyclones Ana and Freddy left widespread destruction in the healthcare sector in all aspects of service delivery. Between road damage, the washing away of bridges, and prolonged flooding at the facilities, the situation left communities with little or no access to care. Participants were concerned with the experience of a total cut in health services due to facility damage caused by floods. A hospital attendant described the aftermath of Cyclone Freddy: “It was flooding all over the place for about three days, and patients were struggling to find help because there was nothing happening here in the health center, as water was everywhere.” (040924TCMHC1)

The trail of destruction left by both cyclones on the roads was reported to cause medicine supply disruptions. One healthcare worker described the challenge: “[In] both Cyclone Ana and Cyclone Freddy, most of our roads were impassable… The district was failing to transport essential commodities, medicines and other supplies, and specifically cholera supplies, to the facility, because the bridge had been washed away.” (170924SNB4)

The growing number of IDPs created additional pressure on health services, which were required to respond to cholera outbreaks and physical injuries while maintaining routine services. The few clinical workers who were working in each facility, as well as the support staff, had to navigate between routine care, such as baby deliveries, to opening cholera treatment wards, and operating mobile clinics in IDP camps. The overwhelming number of tasks for the already limited staff resulted in healthcare workers providing care seven days a week, and in the reduction of non-emergency services, which contributed to compromised quality of care and scope of services for communities.

### The social and economic determinants of health pathway

The devastating cholera transmission occurred against a backdrop of pre-existing social and economic vulnerabilities that created fertile conditions for disease spread. The Southern Region, where both study districts are located, was already recognized as the poorest region in the country prior to the cyclones. Most houses in these rural areas are constructed from mud-bricks, significantly affecting their durability during cyclones and floods [23]. An interviewee described the interconnections between pre-existing social vulnerabilities and the cyclones: “There have been several effects [of these cyclones]...It has, you know, accelerated further economic challenges, social and economic challenges, because these situations hit people who are already challenged.” (190824SNB3)

Within districts and even villages, disaster vulnerabilities differed between populations, distinguishing those who could afford better and more resilient housing and toilet structures from those who could not, mostly along gender and age lines. A nurse from Neno explained:

I would say [that] those living in houses that are not properly constructed, living along the riverbanks – those kinds of places are the ones that are more susceptible. And the elderly, or single women with children and all that, most of the time they are not well taken care of. They easily fall victim to this kind of thing. (140824ANL1)

The intensity of Cyclone Ana forced about 90,000 households in Chikwawa alone to relocate to improvised camps. The harsh conditions in the IDP camps were perceived by study participants as a critical factor contributing to cholera transmission, as people lost not only their houses, but also their kitchen utensils, water buckets, and soap, all essential for preventing infectious diseases. Due to the limited capacity and the large, displaced population, WASH interventions lagged:

We’re talking about an inadequacy of buckets, where we would only have maybe some two or three buckets placed. But the camp had about 200 people [in it]. So, you can just imagine. We talked about toilets. So we put in temporary toilets. So, like in the [camp], we had two. You can just imagine two against that whole population. It was just very hard. (040924SCM2)

Cyclones compromised water as boreholes contaminated with floodwater or were destroyed completely, forcing people to turned to rivers and streams. This, combined with mass destruction of agricultural lands and housing eventuated in people adopting coping strategies that, in lieu of other options, were perceived as contributing to disease transmission pathways. Men along the border with Mozambique took advantage of the slightly stronger currency on the other side of the border to look for piece work in farming or construction. Conversely, political instability in Mozambique drove people to seek asylum in Malawi. A clinical officer from Chikwawa described how cross-border movement also facilitated disease transmission:

A lot of patients come from Mozambique, a far-away place. So, they used to get infected a lot, and had diarrhea a lot. So, when Malawians passed by, they used to get these cholera bacteria. And then it also affected the home around, on this side. Once they come, our clients, our people, when they go to the other side, they get cholera there and then introduce it in Malawi. (030924SCD1)

The cholera outbreak did not know borders, nor did the people who lived on these porous borders. When cholera incidence was low in Malawi, it was difficult to maintain any previously made gains, as movement continued and migrant workers often returned home sick to their communities. The cross-border disease transmission emerged as a central theme in interviews conducted in Chikwawa, as providers felt that they lacked control over these transmission pathways.

### Pathway convergence: The perfect storm for cholera transmission

With this paper, we have illustrated how each of these perceived pathways could potentially stand alone as a driver for cholera transmission. However, it was their convergence that amplified what might have been an annual localized epidemic into the most severe cholera outbreak in Malawi’s history. A healthcare provider summarized how these compounding effects contributed to outbreak dynamics:

One – they have unsafe water. Two – they don’t have adequate food. And so, [there are] issues of diarrhea. And also, on the other hand, most of them, they don’t have pit latrines at home. So, when you combine all these things – there is no food, no potable water, no good waste management – then, issues of diarrhea are very common. (220824SCB1)

The feedback loops between these pathways contributed to further amplification effects. The destruction of healthcare facilities hindered efforts to mobilize disease control and treatment measures, and the destruction of road infrastructure limited access to medical supplies, WASH, and other crucial aid for both displaced populations and communities who remained in their villages. The need to go beyond service delivery in existing healthcare facilities, and extend outreach to the IDP camps, together with dedicating a percentage of the staff to treating only cholera patients in infectious wards or tents, put the system under immense pressure. Additionally, the intersection of acute climate impacts, in the form of successive cyclones, with chronic structural vulnerabilities, generated coping mechanisms that facilitated disease transmission and added additional burdens to the health system and the communities.

The outbreak was officially declared over in July 2024, more than two years after it was declared in early 2022, two months after Cyclone Ana struck the Southern Region [44]. However, the underlying structural vulnerabilities, including in the health system, the deteriorating infrastructure, and declining water and sanitation coverage, remained largely unchanged.

## Discussion

Globally, climate change has increased the burden of infectious diseases, as exemplified by the 2022–2204 cholera outbreak in Malawi [10]. However, climate change, and its manifestation in acute events such as Cyclones Ana and Freddy, cannot alone explain the unprecedented length and scope of this devastating outbreak. Rather, the interconnectedness of different pathways created cascading risks that contributed to this vast impact [45]. The convergence of social, ecological, and health system pathways transformed what healthcare providers historically managed as predictable, seasonal cholera cases into an uncontrollable epidemic that lasted over two years.

The complexity of these pathways, and the feedback loops between them, necessitates moving beyond linear thinking of proximate causation of outbreaks to a more nuanced, in-depth understanding of how global and local structural and social factors shape epidemic dynamics [46–48]. As Giles-Vernick and Villa [16] argue: “Pathogens are the proximate causes of epidemics, but biological and social life are inextricably intertwined, so that social, political economic, ecological conditions and relations profoundly shape epidemic burdens and consequences” (p.3). This shift has implications for achieving ambiguous goals such as cholera elimination in endemic countries by 2030, and for improving disaster preparedness and health systems adaptation, according to the Sendai Framework and the Sustainable Development Goals [2,49].

Although most of the literature already acknowledges ecological, clinical, and microbiological infectious disease pathways, including even complex ones of water-human-pathogen interactions, or One Health approaches that integrate human, animal, and environmental factors, there remains a gap in social epidemiology, of how we understand the structural and societal pathways involved in emergency situations, moving beyond classical epidemiological triangulars [8,46,50,51]. This kind of nuanced understanding is crucial for learning from the real-life experiences of communities and first responders in terms of how to navigate these feedback loops and the new forms in which they contribute to the increase in infectious disease outbreaks [13,32].

Our analysis adds another layer of interconnected social pathways to the Complex Adaptive Systems (CAS) framework recently developed by Talukder et al. [21], which assesses the complex network of health impacts associated with climate change, including integrated effects on communities and healthcare services during health emergencies. By relying on insights from first responders, our analysis exemplifies real outbreaks and validates this framework. Integrating these pathways into a complete, complex picture of emerging outbreaks following climate-related disasters is essential for developing effective cross-sectoral preparedness and adaptation strategies. This shift to complex system thinking helps us avoid “a narrow assessment of the risks” [45].

Furthermore, although there is compelling evidence that climate change, together with insufficient WASH infrastructures, significantly contributes to cholera incidence, these relations do not explain all and depend on local realities and structural and societal factors as well. For example, the Koua et al. analysis of cholera burden in the WHO African region shows that although steps are being taken in order to eliminate cholera by 2030, the complexity of pathway interactions raises questions about their effectiveness [5].

There exists a continental framework for cholera control and elimination, yet implementation remains inconsistent, particularly in addressing the structural vulnerabilities that enable disease transmission during climate-related disasters [5]. A recent assessment of the WHO-African Region Roadmap 2030 for cholera control following the first five years of implementation categorized Malawi’s progress as insufficient [4]. Why are countries like Malawi only partially implementing this framework? The answer lies not only in the obvious reasons of weakness of national and local systems, the dual burden of disease and the economic situation, but also because these frameworks do not take into account what healthcare providers described as “no room for recovery.”

The only milestone Malawi currently meets is the establishment of a multi-sectoral coordination mechanism. However, when it comes to funded, long-term programming and the actual execution of these cross-sectoral mechanisms, the country’s progress remains insufficient [4]. These weaknesses are consistent with the convergence of pathways and barriers identified in this study, highlighting the critical importance of multi-sectoral management across different timescales. Our findings suggest that effective cholera control is often hindered because transmission is fueled by drivers outside the traditional health system, specifically within the realms of social welfare, disaster management, and physical infrastructure. This necessitates a shift in practice toward a multi-sectoral management approach that treats these external drivers as central to outbreak containment.

Additionally, in the case of Malawi, although one contributing factor to the cholera spread involved the introduction of a new, more contagious strain of Vibrio cholerae [20], research by Okpanachi and his colleagues [12] suggests that the fragility of the Malawian health system played a central role in the outbreak’s severity, extending beyond strain characteristics alone. Therefore, the weakness of the Malawian healthcare system impacted every dimension of the outbreak and its response. This fragility also significantly affected the prioritization of policy prescriptions, as cholera was only one of many competing needs within an already resource-constrained system.

The temporal dimension emerged as particularly critical in provider accounts. Healthcare workers described how the compressed timeframe between successive cyclones prevented recovery and preparedness, creating a cascade where each event was built upon unrecovered health facilities, depleted WASH and transportation infrastructure, and overwhelmed staff. These finding challenge traditional disaster management models that assume that after a disaster there will be a recovery period [52]. Instead, we see that the pathways identified by healthcare providers require a different approach. They described a cyclical pattern of a year-long emergency, during which there are floods and cyclones, that require a transition of the whole system into emergency mode, providing routine services and IDP camp services simultaneously – all with sometimes fewer than five clinical staff per facility. The degraded conditions at camps and in communities following these emergencies, combined with the coping mechanisms available to the communities, contribute to infectious disease outbreaks, necessitating additional manpower to control the outbreak and treat patients. With limited resources and limited global attention, authorities and humanitarian actors tend to focus on the immediate response, with fewer resources allocated to recovery.

Although the short-term pathway of the disaster includes the conditions that contribute to infection, it is the long-term pathway that matters most – the ability to recover infrastructure, resettle IDPs, and regain the normal tempo of the health system [8]. Without these aspects, as demonstrated following Cyclone Ana in Malawi, the partially repaired systems remain vulnerable to subsequent climate events. Cyclone Freddy exemplified this vulnerability, perpetuating conditions conducive to future outbreaks [33]. This temporal complexity is compounded by increasing unpredictability in disease and weather patterns. Even when time permits better preparation, projecting disease spread and extreme weather events has become increasingly complex and often not possible [53].

Another challenge to implementing the cholera elimination framework is connected to cross-border transmission dynamics, as identified by health workers. Their accounts of differential outbreak thresholds between Malawi and Mozambique, combined with economic migration patterns intensified by agricultural destruction, demonstrate how climate impacts combine with social marginality, creating transnational disease transmission networks that transcend existing control frameworks. These findings suggest that elimination efforts require regional coordination mechanisms that can address the structural drivers of cross-border movement, rather than focusing solely on surveillance and case management [19,54].

The complexity of pathway interactions raises fundamental questions about the feasibility of current cholera elimination timelines and strategies [54–56]. When healthcare systems are overwhelmed by competing priorities, limited resources, and staff shortages, temporary WASH interventions alone cannot fully control disease spread. Although Sack [57] argued that oral cholera vaccines represented a “game changer” in the quest to eradicate cholera, focusing solely on vaccination, better projections, and surveillance may not be sufficient given the interconnected nature of outbreak pathways. Healthcare workers’ emphasis on the unpredictability of current weather and disease patterns indicates that elimination strategies must account for the ways climate change is disrupting established disease control assumptions.

### Policy recommendations

These insights point toward the need to address the interconnected nature of these pathways in order to both reduce population vulnerability and create better services and infrastructures [9,21], aligning with disaster management approaches that emphasize top-hazard or all-hazards frameworks [58].

In the short-term, initial steps should focus on operational preparedness and immediate logistical constraints. These include adding more staff before rainy seasons, improving communication around the border, and stockpiling supplies at health facilities likely to be isolated because of bad roads.

In the medium-term, system improvements should focus on workforce stability and a deeper integration of frontline needs into disaster management. This involves increasing permanent staffing levels and investing in the specific training and tools required for healthcare teams to handle concurrent extreme weather events and outbreaks. Furthermore, developing improved early warning systems for both weather and emerging diseases is essential to provide teams with the predictive data necessary to anticipate these threats. Learning from frontline workers what the challenges are in handling extreme weather events and outbreaks can help improve preparedness and investment in what can make the most impact.

In the long-term, system improvement, in the form of more staff and a better understanding of the needs of the affected population and the teams that provide for them [27,32], is vital not only for coping better with climate change and infectious diseases, but for improving life conditions and health outcomes. However, without addressing the root causes by building resilient WASH infrastructure in all villages, waterborne diseases cannot be truly eradicated [59]. While multisectoral management mechanisms exist [4], they require sustained programming, comprehensive social mobilization strategies, and stable, long-term investments in case management capacity and infrastructure to be effective.

### Study limitation

Healthcare and emergency workers represent the frontline of climate change adaptation, poverty alleviation, and emerging health threats, continuously adapting through necessity. However, in this paper we focused on describing and analyzing climate-induced disease outbreaks’ pathways. Future research should examine provider adaptations and navigation strategies in greater detail. Moreover, although the premise of this article lies in the importance of the role played by healthcare workers, their perspectives should be complemented by those of other stakeholders, including of community members, civil servants from other sectors, NGOs, and traditional authorities. Additional research is needed in order to understand perceived transmission pathways in areas less affected by Cyclones Ana and Freddy, including the Northern and Central Regions, as our findings are deeply contextualized within the socio-political landscape of the Southern Region. This context also limits the transferability of these results to urban settings.

The focus of the study’s insights on one country from a purely qualitative perspective limits the generalizability of the findings and may require adaptations when mapping social and cultural pathways of disease outbreaks in other parts of the world. Furthermore, this study analyzed the experiences of first responders, which cannot account for the full breadth of the outbreak nor the complete range of perspectives across different sectors. Their narratives were inevitably influenced by their relationship with PIH/APZU and their specific involvement in the response to Cyclones Ana and Freddy. While we sought to mitigate these limitations through data saturation, multiple field visits and observations, and the reflexivity strategies previously described, the findings remain an interpretation of specific experiences within a unique socio-political context.

It is also important to note that these qualitative insights are not intended to provide epidemiologic proof of causation. Instead, they offer a systems-thinking perspective on multi-drivers and sectors convergence and their critical manifestation in cholera outbreaks. Understanding the interconnectedness between healthcare systems and social, economic, and environmental pathways is therefore essential for developing effective adaptation and intervention strategies that can address the complex realities of climate-induced disease outbreaks.

Tracking the social and cultural pathways of these outbreaks, our findings indicate that taken together, the factors shaping the outbreaks go beyond pathogens and cyclones and include workforce shortages, insufficient WASH infrastructures, and the overall weakness of the healthcare system, especially in rural and remote areas. By focusing on social and cultural pathways of cholera outbreaks and their intricate and interconnected nature, this study contributes to a better understanding of the failures of existing cholera control measures. These insights extend beyond cholera outbreaks in Malawi and have practical implications for the design of outbreak control responses worldwide.

Overall, this study underscores the importance of mapping, understanding, and addressing the social and cultural pathways of disease outbreak, ultimately leading to stronger and more effective measures for climate-induced disease outbreaks.

## Supporting information

S1 File COREQ (COnsolidated criteria for REporting Qualitative research) Checklist. (PDF)
