# Supplementary material for: The convergence of health system, climate, and social pathways in Malawi’s 2022–2024 cholera outbreak – A qualitative assessment
Source: PLoS Negl Trop Dis. 2026 Jul 10;20(7):e0014529. doi: 10.1371/journal.pntd.0014529 (PMC13375130; doi:10.1371/journal.pntd.0014529)
Supplement: Attachment — Submitted filename: Cholera_response to reviewers.docx [file pntd.0014529.s003.docx]

**PNTD-D-25-02247 - The Convergence of Health System, Climate, and Social Pathways in Malawi's 2022-2024 Cholera Outbreak - A Qualitative Assessment**

**Point by point response:**

**Reviewer #1**

**Comment #1:** "First, the authors are requested to clarify the meaning of the phrase “removed for review,” which appears in the sections where the ethics committee identifiers should be included. It is important to specify whether this phrase was used as a placeholder during the anonymized peer-review process. If so, the final version of the manuscript should explicitly include the names or identifiers of all ethics committees..."; "Similarly, in line 142, the sentence “The current study was conducted in collaboration with (Removed for review)” requires clarification. The authors should replace this placeholder with the name of the collaborating institution or organization."

- **Response #1:** These were placeholders for a double-blind review. The names have now appeared in the version for reviewers as well.
- **Location in Manuscript:** Methods: p. 5, p. 8.

**Comment #2:** "Additionally, the manuscript should address the potential implications of having participants with pre-existing working relationships with members of the study team... for example, through social desirability bias, response bias, or power dynamics. A brief reflection on how this issue was mitigated... would strengthen the study's methodological rigor."

- **Response #2:** This is an important point. The pre-existing relationships between certain team members and the study population facilitated better access and encouraged participants to openly share their experiences, as they already maintained a level of trust with the collaborating organization. However, we recognize that these relationships naturally carry inherent power dynamics and institutional politics within the healthcare system. Therefore, we expended the Reflexivity Statement to address this comment. Specifically, we detailed how we mitigated these biases by ensuring that all interviews were conducted by researchers who were external to the partner organization, maintaining strict participant anonymity, and engaging in continuous team debriefing and reflexivity sessions to ensure that the findings were grounded in the collected data.
- **Location in Manuscript:** Methods, pp. 8-9.

**Comment #3:** "While these findings are valuable, the discussion would benefit from a broader perspective. In particular, it would be important to incorporate an analysis of the role of multisectoral participation in cholera prevention and control, as well as the importance of political will in implementing effective public health interventions."

- **Response #3:** We would like to thank the reviewer for this crucial comment. We have expanded the discussion to better analyze the role of multisectoral participation and political will in light of recent assessments of the WHO Roadmap 2030 in the African Region, addressing the limited progress made by Malawi. We have amended the argument to include the observation that effective cholera control is frequently hindered because transmission is fueled by drivers outside the traditional healthcare system, as evidenced by our analysis and the existing literature. Furthermore, we address the comment on political will by discussing how the inherent fragility of the healthcare system forces a difficult prioritization of policy prescriptions, where cholera must compete for resources against a multitude of other acute needs.
- **Location in Manuscript:** Discussion, pp. 17-18.

**Comment #4:** "Finally, the manuscript would benefit from a more robust discussion of its limitations from a qualitative research perspective... reflect on aspects such as the contextual nature of the findings... potential influence of researcher positionality... and issues such as sample diversity, data saturation, and reflexivity."

- **Response #4:** We revised the Reflexivity Statement to include study limitation of our positionality and qualitative methods and have significantly expanded the Limitations section to address these qualitative considerations.
- **Location in Manuscript:** Methods, pp. 8-9, Discussion, pp. 19-20.

**Reviewer #2**

**Comment #1:** "The manuscript repeatedly states that previous cholera research has focused mainly on biomedical or epidemiological approaches and neglected system interactions. There is already substantial literature on disease transmission, WASH systems, disaster vulnerability, and One Health frameworks... The authors should explain the motivation and rationale for this study in the introduction."

- **Response #1:** We thank the reviewer for this constructive feedback and for providing these relevant references, especially Kita et al.’s (2026) paper. We acknowledge the established literature, as reflected in the revised introduction and discussion sections. While many frameworks acknowledge ecological and clinical pathways or use One Health approaches, there remains a gap in understanding context-specific structural and societal pathways of cascading disasters. This is especially true regarding the source of the analysis; in most cases, existing research focuses on policy documents and higher-level policymakers. Our study is motivated by the need to offer an integrated system approach that captures the multifaceted nature of cholera transmission in disaster context from below. A key rationale for this work is that healthcare provider perspectives on these pathway interconnections remain largely unexplored. By focusing on these frontline insights, we complement high-level policy research, such as Kita et al.’s (2026) work on systemic risks during the COVID-19 pandemic in Malawi. The Introduction has been revised to better situate these motivations within the existing literature.
- **Location in Manuscript:** Introduction, p. 3, p. 5.

**Comment #2:** "The participant numbers in the 'Education Level' section of Table 1 for Neno District, the sample size is listed as 12, yet the education rows sum to 18 (Secondary: 1, Diploma: 5, Higher Education: 12). Please explain this."

- **Response #2:** We thank the reviewer for identifying this discrepancy. The values in the columns for the "Education Level" section of Table 1 were inadvertently transposed during formatting. We have corrected the table.
- **Location in Manuscript:** Table 1, pp. 7-8.

**Comment #3:** "The authors interviewed 24 'first responders' across two districts, but this group appears heterogeneous... It is unclear whether this sample adequately captures the breadth of actors involved in outbreak response. Please explain: Why only these two districts? Why exclude community health workers, NGOs, patients, traditional leaders, WASH officers, or community members?"

- **Response #3:** We thank the reviewer for this important question regarding our study population and setting. We focused our research on Neno and Chikwawa Districts because they were severely impacted by the successive impacts of Cyclones Ana and Freddy and the subsequent cholera outbreaks. While these two districts share a geographic region, they offer distinct complexities in terms of size, accessibility, infrastructure, the cyclones’ impact, and their landscape, allowing for a diverse assessment of the challenges.

Regarding the heterogeneity of the participants, we argue that first responders in rural Malawi are inherently a small but very diverse group. Our sample explicitly included clinical officers, nurses, doctors, environmental health officers, community health workers (health surveillance assistants) and facility managers in small to medium health centers to capture this internal diversity within the healthcare system. While we acknowledge that there are other important actors in cholera prevention and control, we intentionally focused on the perspectives of formal health workers. We elaborated on these choices in the Methods section.

- **Location in Manuscript:** Methods, pp. 5-7, Discussion, p. 20.

**Comment #4:** "The manuscript sometimes implies causal conclusions... However, the data are based on perceptions from 24 interviews, not epidemiologic causal inference... Please revise language to distinguish among participant perceptions, hypothesis-generating interpretations and established epidemiologic evidence."

- **Response #4:** We thank the reviewer for this insightful comment. Our study does not intend to provide epidemiologic causal inference but rather to use qualitative descriptive methods to identify how first responders perceive the convergence of complex pathways during an outbreak. We have carefully revised the manuscript to ensure that our language reflects this distinction.
- **Location in Manuscript:** Result, p. 9, pp. 13-15, Conclusions, p. 20.

**Comment #5:** "Policy recommendations are broad... Please prioritize feasible short-, medium-, and long-term actions for Malawi or similar settings."

- **Response #5:** We thank the reviewer for this great suggestion to prioritize our recommendations based on short-, medium-, and long-term actions. We have revised the Policy Recommendations section accordingly. The updated section explicitly categorizes feasible interventions into short-term (e.g., surge staffing and pre-positioning supplies), medium-term (e.g., specialized training), and long-term goals (e.g., resilient WASH infrastructure and multisectoral programming).
- **Location in Manuscript:** Discussion, pp. 19-20.

**Comment #6:** "Please mention the limitations of this study in the discussion section."

- **Response #6:** We have expanded the Limitations section and added a dedicated subsection heading to improve the structural clarity of the manuscript.
- **Location in Manuscript:** Discussion, pp. 19-20
