# Supplementary material for: The convergence of health system, climate, and social pathways in Malawi’s 2022–2024 cholera outbreak – A qualitative assessment
Source: PLoS Negl Trop Dis. 2026 Jul 10;20(7):e0014529. doi: 10.1371/journal.pntd.0014529 (PMC13375130; doi:10.1371/journal.pntd.0014529)
Supplement: Attachment — Submitted filename: letter_cholera_100626.docx [file pntd.0014529.s004.docx]

June 12th, 2026

Dear Dr. Phillips, Dr. Picardeau, and the editorial team,

Re: Revised manuscript titled "*The Convergence of Health System, Climate, and Social Pathways in Malawi's 2022-2024 Cholera Outbreak - A Qualitative Assessment"* (ID PNTD-D-25-02247) resubmission to *PLOS Neglected Tropical Diseases*

Thank you for the opportunity to revise our manuscript for *PLOS Neglected Tropical Diseases (PLOS NTD)*.

We have now addressed all the following journal requirements:

1. We have uploaded to and processed all figures by the PLOS NAAS tool. We have also added a link for the base map of figure 1.
2. We have formatted the references and citations based on PLOS NTD citation style.
3. We have reviewed and adjusted the manuscript to strictly adhere to the PLOS NTD style and layout guidelines.

We look forward to your further assessment of our manuscript.

Kind regards,

Shahar Livne

Corresponding author

Ben-Gurion University of the Negev

[Shalivne@gmail.com](mailto:Shalivne@gmail.com)
