# Supplementary material for: The convergence of health system, climate, and social pathways in Malawi’s 2022–2024 cholera outbreak – A qualitative assessment
Source: PLoS Negl Trop Dis. 2026 Jul 10;20(7):e0014529. doi: 10.1371/journal.pntd.0014529 (PMC13375130; doi:10.1371/journal.pntd.0014529)
Supplement: Peer review history

# Peer review: Decision Letter 0

**DOI:** 10.1371/journal.pntd.0014529.r001

**Authors:** Mathieu Picardeau, Richard Phillips

27 Apr 2026

PNTD-D-25-02247

The Convergence of Health System, Climate, and Social Pathways in Malawi's 2022-2024 Cholera Outbreak - A Qualitative Assessment

PLOS Neglected Tropical Diseases

Dear Dr. Livne,

Thank you for submitting your manuscript to PLOS Neglected Tropical Diseases. After careful consideration, we feel that it has merit but does not fully meet PLOS Neglected Tropical Diseases's publication criteria as it currently stands. Therefore, we invite you to submit a revised version of the manuscript that addresses the points raised during the review process.

Please submit your revised manuscript by Jun 26 2026 11:59PM. If you will need more time than this to complete your revisions, please reply to this message or contact the journal office at plosntds@plos.org. When you're ready to submit your revision, log on to https://www.editorialmanager.com/pntd/ and select the 'Submissions Needing Revision' folder to locate your manuscript file.

Please include the following items when submitting your revised manuscript:

* A letter that responds to each point raised by the editor and reviewer(s). You should upload this letter as a separate file labeled 'Response to Reviewers'. This file does not need to include responses to any formatting updates and technical items listed in the 'Journal Requirements' section below.

* A marked-up copy of your manuscript that highlights changes made to the original version. You should upload this as a separate file labeled 'Revised Manuscript with Track Changes'.

* An unmarked version of your revised paper without tracked changes. You should upload this as a separate file labeled 'Manuscript'.

If you would like to make changes to your financial disclosure, competing interests statement, or data availability statement, please make these updates within the submission form at the time of resubmission. Guidelines for resubmitting your figure files are available below the reviewer comments at the end of this letter.

We look forward to receiving your revised manuscript.

Kind regards,

Richard Odame Phillips

Academic Editor

PLOS Neglected Tropical Diseases

-->Mathieu Picardeau

Section Editor

PLOS Neglected Tropical Diseases

Shaden Kamhawi

co-Editor-in-Chief

PLOS Neglected Tropical Diseases

orcid.org/0000-0003-4304-636XX

Paul Brindley

co-Editor-in-Chief

PLOS Neglected Tropical Diseases

orcid.org/0000-0003-1765-0002

**Journal Requirements:**

If the reviewer comments include a recommendation to cite specific previously published works, please review and evaluate these publications to determine whether they are relevant and should be cited. There is no requirement to cite these works unless the editor has indicated otherwise.

1) Please provide an Author Summary. This should appear in your manuscript between the Abstract (if applicable) and the Introduction, and should be 150-200 words long. The aim should be to make your findings accessible to a wide audience that includes both scientists and non-scientists. Sample summaries can be found on our website under Submission Guidelines:

https://journals.plos.org/plosntds/s/submission-guidelines#loc-parts-of-a-submission

2) Please upload all main figures as separate Figure files in .tif or .eps format. For more information about how to convert and format your figure files please see our guidelines:

https://journals.plos.org/plosntds/s/figures

3) We have noticed that you have uploaded Supporting Information files, but you have not included a list of legends. Please add a full list of legends for your Supporting Information files after the references list.

4) We note that you have indicated that there are restrictions to data sharing for this study. PLOS only allows data to be available upon request if there are legal or ethical restrictions on sharing data publicly. For more information on unacceptable data access restrictions, please see https://journals.plos.org/plosntds/s/data-availability#loc-unacceptable-data-access-restrictions.

Before we proceed with your manuscript, please address the following prompts:

a) If there are ethical or legal restrictions on sharing a de-identified data set, please explain them in detail (e.g., data contain potentially identifying or sensitive patient information, data are owned by a third-party organization, etc.) and who has imposed them (e.g., a Research Ethics Committee or Institutional Review Board, etc.). Please also provide contact information for a data access committee, ethics committee, or other institutional body to which data requests may be sent.

b) If there are no restrictions, please upload the minimal anonymized data set necessary to replicate your study findings to a stable, public repository and provide us with the relevant URLs, DOIs, or accession numbers. For a list of recommended repositories, please see https://journals.plos.org/plosone/s/recommended-repositories. You also have the option of uploading the data as Supporting Information files, but we would recommend depositing data directly to a data repository if possible.

We will update your Data Availability statement on your behalf to reflect the information you provide.

5) Please amend your detailed Financial Disclosure statement. This is published with the article. It must therefore be completed in full sentences and contain the exact wording you wish to be published.

1) State the initials, alongside each funding source, of each author to receive each grant. For example: "This work was supported by the National Institutes of Health (####### to AM; ###### to CJ) and the National Science Foundation (###### to AM)."

2) State what role the funders took in the study. If the funders had no role in your study, please state: "The funders had no role in study design, data collection and analysis, decision to publish, or preparation of the manuscript."

3) If any authors received a salary from any of your funders, please state which authors and which funders..

If you did not receive any funding for this study, please simply state: u201cThe authors received no specific funding for this work.u201d

6) Some material included in your submission may be copyrighted. According to PLOS’s copyright policy, authors who use figures or other material (e.g., graphics, clipart, maps) from another author or copyright holder must demonstrate or obtain permission to publish this material under the Creative Commons Attribution 4.0 International (CC BY 4.0) License used by PLOS journals. Please closely review the details of PLOS’s copyright requirements here: PLOS Licenses and Copyright. If you need to request permissions from a copyright holder, you may use PLOS's Copyright Content Permission form.

Please respond directly to this email and provide any known details concerning your material's license terms and permissions required for reuse, even if you have not yet obtained copyright permissions or are unsure of your material's copyright compatibility. Once you have responded and addressed all other outstanding technical requirements, you may resubmit your manuscript within Editorial Manager.

Potential Copyright Issues:

i) Figure 1: please (a) provide a direct link to the base layer of the map (i.e., the country or region border shape) and ensure this is also included in the figure legend; and (b) provide a link to the terms of use / license information for the base layer image or shapefile. We cannot publish proprietary or copyrighted maps (e.g. Google Maps, Mapquest) and the terms of use for your map base layer must be compatible with our CC BY 4.0 license.

Note: if you created the map in a software program like R or ArcGIS, please locate and indicate the source of the basemap shapefile onto which data has been plotted.

If your map was obtained from a copyrighted source please amend the figure so that the base map used is from an openly available source. Alternatively, please provide explicit written permission from the copyright holder granting you the right to publish the material under our CC BY 4.0 license.

If you are unsure whether you can use a map or not, please do reach out and we will be able to help you. The following websites are good examples of where you can source open access or public domain maps:

* U.S. Geological Survey (USGS) - All maps are in the public domain. (http://www.usgs.gov)

* PlaniGlobe - All maps are published under a Creative Commons license so please cite “PlaniGlobe, http://www.planiglobe.com, CC BY 2.0” in the image credit after the caption. (http://www.planiglobe.com/?lang=enl)

* Natural Earth - All maps are public domain. (http://www.naturalearthdata.com/about/terms-of-use/)

7) Please send a completed 'Competing Interests' statement, including any COIs declared by your co-authors. If you have no competing interests to declare, please state "The authors have declared that no competing interests exist". Otherwise please declare all competing interests beginning with the statement "I have read the journal's policy and the authors of this manuscript have the following competing interests"

**Reviewers' comments:**

Reviewer's Responses to Questions

**Key Review Criteria Required for Acceptance?**

As you describe the new analyses required for acceptance, please consider the following:

**Methods**

-Are the objectives of the study clearly articulated with a clear testable hypothesis stated?

-Is the study design appropriate to address the stated objectives?

-Is the population clearly described and appropriate for the hypothesis being tested?

-Is the sample size sufficient to ensure adequate power to address the hypothesis being tested?

-Were correct statistical analysis used to support conclusions?

-Are there concerns about ethical or regulatory requirements being met?

Reviewer #1: The manuscript addresses a topic that is relevant and of clear interest to the journal's readership. The focus on the determinants and patterns associated with cholera outbreaks contributes meaningfully to the current body of knowledge, particularly in the context of environmental and systemic drivers of infectious diseases.

However, several aspects of the manuscript would benefit from clarification and further elaboration:

First, the authors are requested to clarify the meaning of the phrase “removed for review,” which appears in the sections where the ethics committee identifiers should be included. It is important to specify whether this phrase was used as a placeholder during the anonymized peer-review process. If so, the final version of the manuscript should explicitly include the names or identifiers of all ethics committees that reviewed and approved the study, in accordance with ethical reporting standards.

Similarly, in line 142, the sentence “The current study was conducted in collaboration with (Removed for review)” requires clarification. The authors should replace this placeholder with the name of the collaborating institution or organization. Transparency in institutional collaboration is essential for assessing the research context and credibility.

Additionally, the manuscript should address the potential implications of having participants with pre-existing working relationships with members of the study team. The authors are encouraged to discuss whether this circumstance could have influenced the responses obtained during interviews, for example, through social desirability bias, response bias, or power dynamics. A brief reflection on how this issue was mitigated—or why it is unlikely to have significantly affected the findings—would strengthen the study's methodological rigor.

The authors identify three main patterns as major causes of cholera outbreaks: growing unpredictability, the role of extreme weather events as disease catalysts, and service disruptions caused by infrastructure damage. While these findings are valuable, the discussion would benefit from a broader perspective. In particular, it would be important to incorporate an analysis of the role of multisectoral participation in cholera prevention and control, as well as the importance of political will in implementing effective public health interventions. These dimensions are critical for translating findings into actionable policy and practice.

Finally, the manuscript would benefit from a more robust discussion of its limitations from a qualitative research perspective. The authors are encouraged to reflect on aspects such as the contextual nature of the findings, which may limit their transferability to other settings; the potential influence of researcher positionality and interpretation during data collection and analysis; and the reliance on self-reported data, which may be shaped by participants’ perceptions and experiences. Additionally, discussing issues such as sample diversity, data saturation, and reflexivity would enhance the study's transparency and rigor. Acknowledging these elements would strengthen the credibility and trustworthiness of the findings.

Reviewer #2: (No Response)

**********

**Results**

-Does the analysis presented match the analysis plan?

-Are the results clearly and completely presented?

-Are the figures (Tables, Images) of sufficient quality for clarity?

Reviewer #1: please see above

Reviewer #2: (No Response)

**********

**Conclusions**

-Are the conclusions supported by the data presented?

-Are the limitations of analysis clearly described?

-Do the authors discuss how these data can be helpful to advance our understanding of the topic under study?

-Is public health relevance addressed?

Reviewer #1: please see above

Reviewer #2: (No Response)

**********

**Editorial and Data Presentation Modifications?**

Use this section for editorial suggestions as well as relatively minor modifications of existing data that would enhance clarity. If the only modifications needed are minor and/or editorial, you may wish to recommend “Minor Revision” or “Accept”.

Reviewer #1: please see above

Reviewer #2: (No Response)

**********

**Summary and General Comments**

Use this section to provide overall comments, discuss strengths/weaknesses of the study, novelty, significance, general execution and scholarship. You may also include additional comments for the author, including concerns about dual publication, research ethics, or publication ethics. If requesting major revision, please articulate the new experiments that are needed.

Reviewer #1: please see above

Reviewer #2: This manuscript offers an insightful qualitative analysis of the factors affecting the 2022-2024 cholera outbreak in Malawi. By employing a systems-thinking approach, it presents valuable perspectives that go beyond traditional biomedical models, highlighting crucial insights for enhancing disaster preparedness and achieving disease elimination goals.

I have the following comments\suggestions that need to be taken care of:

1. The manuscript repeatedly states that previous cholera research has focused mainly on biomedical or epidemiological approaches and neglected system interactions. There is already substantial literature on disease transmission, WASH systems, disaster vulnerability, and One Health frameworks (see, for example, https://doi.org/10.1111/risa.70229, 10.3389/fpubh.2026.1755792, 10.3934/publichealth.2025039). The authors should explain the motivation and rationale for this study in the introduction.

2. The participant numbers in the "Education Level" section of Table 1 for Neno District, the sample size is listed as 12, yet the education rows sum to 18 (Secondary: 1, Diploma: 5, Higher Education: 12). Please explain this.

3. The authors interviewed 24 "first responders" across two districts, but this group appears heterogeneous (clinical staff, environmental staff, management, others). It is unclear whether this sample adequately captures the breadth of actors involved in outbreak response. Please explain: Why only these two districts? Why exclude community health workers, NGOs, patients, traditional leaders, WASH officers, or community members?

4. The manuscript sometimes implies causal conclusions (e.g., cyclones transformed seasonal cholera into a two-year epidemic; pathway convergence caused outbreak magnitude). However, the data are based on perceptions from 24 interviews, not epidemiologic causal inference. Qualitative data are powerful for understanding mechanisms and lived systems' experiences, but are not sufficient alone to prove causation. Please revise language to distinguish among participant perceptions, hypothesis-generating interpretations and established epidemiologic evidence.

5. Policy recommendations are broad (more staff, stockpiling, resilient WASH, border coordination, systems thinking). Please prioritize feasible short-, medium-, and long-term actions for Malawi or similar settings.

6. Please mention the limitations of this study in the discussion section.

**********

PLOS authors have the option to publish the peer review history of their article (what does this mean?). If published, this will include your full peer review and any attached files.

If you choose “no”, your identity will remain anonymous but your review may still be made public.

**Do you want your identity to be public for this peer review?**  For information about this choice, including consent withdrawal, please see our Privacy Policy.

Reviewer #1: No

Reviewer #2: No

**Figure resubmission:**

While revising your submission, we strongly recommend that you use PLOS’s NAAS tool (https://ngplosjournals.pagemajik.ai/artanalysis) to test your figure files. NAAS can convert your figure files to the TIFF file type and meet basic requirements (such as print size, resolution), or provide you with a report on issues that do not meet our requirements and that NAAS cannot fix.

After uploading your figures to PLOS’s NAAS tool - https://ngplosjournals.pagemajik.ai/artanalysis, NAAS will process the files provided and display the results in the "Uploaded Files" section of the page as the processing is complete. If the uploaded figures meet our requirements (or NAAS is able to fix the files to meet our requirements), the figure will be marked as "fixed" above. If NAAS is unable to fix the files, a red "failed" label will appear above. When NAAS has confirmed that the figure files meet our requirements, please download the file via the download option, and include these NAAS processed figure files when submitting your revised manuscript.

**Reproducibility:**

To enhance the reproducibility of your results, we recommend that authors of applicable studies deposit laboratory protocols in protocols.io, where a protocol can be assigned its own identifier (DOI) such that it can be cited independently in the future. Additionally, PLOS ONE offers an option to publish peer-reviewed clinical study protocols. Read more information on sharing protocols at https://plos.org/protocols?utm_medium=editorial-email&utm_source=authorletters&utm_campaign=protocols

# Author comment: Author response to Decision Letter 1

**DOI:** 10.1371/journal.pntd.0014529.r002

30 May 2026

Attachment Submitted filename: Cholera_response to reviewers.docx

# Editor report: Decision Letter 1

**DOI:** 10.1371/journal.pntd.0014529.r003

**Authors:** Mathieu Picardeau, Richard Phillips, Mathieu Picardeau, Richard Phillips

10 Jun 2026

PNTD-D-25-02247R1

The Convergence of Health System, Climate, and Social Pathways in Malawi's 2022-2024 Cholera Outbreak - A Qualitative Assessment

PLOS Neglected Tropical Diseases

Dear Dr. Livne,

Thank you for submitting your manuscript to PLOS Neglected Tropical Diseases. After careful consideration, we feel that it has merit but does not fully meet PLOS Neglected Tropical Diseases's publication criteria as it currently stands. Therefore, we invite you to submit a revised version of the manuscript that addresses the points raised during the review process.

Please submit your revised manuscript by Aug 09 2026 11:59PM. If you will need more time than this to complete your revisions, please reply to this message or contact the journal office at plosntds@plos.org. When you're ready to submit your revision, log on to https://www.editorialmanager.com/pntd/ and select the 'Submissions Needing Revision' folder to locate your manuscript file.

Please include the following items when submitting your revised manuscript:

* A letter that responds to each point raised by the editor and reviewer(s). You should upload this letter as a separate file labeled 'Response to Reviewers'. This file does not need to include responses to any formatting updates and technical items listed in the 'Journal Requirements' section below.

* A marked-up copy of your manuscript that highlights changes made to the original version. You should upload this as a separate file labeled 'Revised Manuscript with Track Changes'.

* An unmarked version of your revised paper without tracked changes. You should upload this as a separate file labeled 'Manuscript'.

If you would like to make changes to your financial disclosure, competing interests statement, or data availability statement, please make these updates within the submission form at the time of resubmission. Guidelines for resubmitting your figure files are available below the reviewer comments at the end of this letter.

As the corresponding author, your ORCID iD is verified in the submission system and will appear in the published article. PLOS supports the use of ORCID, and we encourage all coauthors to register for an ORCID iD and use it as well. Please encourage your coauthors to verify their ORCID iD within the submission system before final acceptance, as unverified ORCID iDs will not appear in the published article. Only the individual author can complete the verification step; PLOS staff cannot verify ORCID iDs on behalf of authors.

We look forward to receiving your revised manuscript.

Kind regards,

Richard Odame Phillips

Academic Editor

PLOS Neglected Tropical Diseases

Mathieu Picardeau

Section Editor

PLOS Neglected Tropical Diseases

Shaden Kamhawi

co-Editor-in-Chief

PLOS Neglected Tropical Diseases

orcid.org/0000-0003-4304-636XX

Paul Brindley

co-Editor-in-Chief

PLOS Neglected Tropical Diseases

orcid.org/0000-0003-1765-0002

**Additional Editor Comments (if provided):**

Thank you for submitting your revised manuscript. However, a few outstanding issues need to be addressed

Formatting: Please ensure that the manuscript adheres strictly to the PLOS NTD formatting guidelines.

Figure legends: Figure legends should be placed appropriately (i.e., after the reference list, with each legend on a separate page, as required by the journal).

References: The reference list does not currently conform to the PLOS NTD (Vancouver) style. Please correct the formatting of all citations throughout the entire manuscript.

We look forward to receiving your revised submission.

**Reviewers' comments:**

-->[NOTE: If reviewer comments were submitted as an attachment file, they will be attached to this email and accessible via the submission site. Please log into your account, locate the manuscript record, and check for the action link "View Attachments". If this link does not appear, there are no attachment files.]

**Figure resubmission:**

While revising your submission, we strongly recommend that you use PLOS’s NAAS tool (https://ngplosjournals.pagemajik.ai/artanalysis) to test your figure files. NAAS can convert your figure files to the TIFF file type and meet basic requirements (such as print size, resolution), or provide you with a report on issues that do not meet our requirements and that NAAS cannot fix.

After uploading your figures to PLOS’s NAAS tool - https://ngplosjournals.pagemajik.ai/artanalysis, NAAS will process the files provided and display the results in the "Uploaded Files" section of the page as the processing is complete. If the uploaded figures meet our requirements (or NAAS is able to fix the files to meet our requirements), the figure will be marked as "fixed" above. If NAAS is unable to fix the files, a red "failed" label will appear above. When NAAS has confirmed that the figure files meet our requirements, please download the file via the download option, and include these NAAS processed figure files when submitting your revised manuscript.

**Reproducibility:**

To enhance the reproducibility of your results, we recommend that authors of applicable studies deposit laboratory protocols in protocols.io, where a protocol can be assigned its own identifier (DOI) such that it can be cited independently in the future. Additionally, PLOS ONE offers an option to publish peer-reviewed clinical study protocols. Read more information on sharing protocols at https://plos.org/protocols?utm_medium=editorial-email&utm_source=authorletters&utm_campaign=protocols

# Author comment: Author response to Decision Letter 2

**DOI:** 10.1371/journal.pntd.0014529.r004

15 Jun 2026

Attachment Submitted filename: letter_cholera_100626.docx

# Editor report: Decision Letter 2

**DOI:** 10.1371/journal.pntd.0014529.r005

**Authors:** Mathieu Picardeau, Richard Phillips, Mathieu Picardeau, Richard Phillips, Mathieu Picardeau, Richard Phillips

30 Jun 2026

Dear Ms Livne,

We are pleased to inform you that your manuscript 'The Convergence of Health System, Climate, and Social Pathways in Malawi's 2022-2024 Cholera Outbreak - A Qualitative Assessment' has been provisionally accepted for publication in PLOS Neglected Tropical Diseases.

Before your manuscript can be formally accepted you will need to complete some formatting changes, which you will receive in a follow up email. A member of our team will be in touch with a set of requests.

Please note that your manuscript will not be scheduled for publication until you have made the required changes, so a swift response is appreciated.

IMPORTANT: The editorial review process is now complete. PLOS will only permit corrections to spelling, formatting or significant scientific errors from this point onwards. Requests for major changes, or any which affect the scientific understanding of your work, will cause delays to the publication date of your manuscript.

Should you, your institution's press office or the journal office choose to press release your paper, you will automatically be opted out of early publication. We ask that you notify us now if you or your institution is planning to press release the article. All press must be co-ordinated with PLOS.

Thank you again for supporting Open Access publishing; we are looking forward to publishing your work in PLOS Neglected Tropical Diseases.

Best regards,

Richard Odame Phillips

Academic Editor

PLOS Neglected Tropical Diseases

Mathieu Picardeau

Section Editor

PLOS Neglected Tropical Diseases

Shaden Kamhawi

co-Editor-in-Chief

PLOS Neglected Tropical Diseases

orcid.org/0000-0003-4304-636XX

Paul Brindley

co-Editor-in-Chief

PLOS Neglected Tropical Diseases

orcid.org/0000-0003-1765-0002

***********************************************************

p.p1 {margin: 0.0px 0.0px 0.0px 0.0px; line-height: 16.0px; font: 14.0px Arial; color: #323333; -webkit-text-stroke: #323333}span.s1 {font-kerning: none

# Editor report: Acceptance letter

**DOI:** 10.1371/journal.pntd.0014529.r006

**Authors:** Mathieu Picardeau, Richard Phillips, Mathieu Picardeau, Richard Phillips, Mathieu Picardeau, Richard Phillips

Dear Ms Livne,

We are delighted to inform you that your manuscript, "The Convergence of Health System, Climate, and Social Pathways in Malawi's 2022-2024 Cholera Outbreak - A Qualitative Assessment," has been formally accepted for publication in PLOS Neglected Tropical Diseases.

We have now passed your article onto the PLOS Production Department who will complete the rest of the publication process. All authors will receive a confirmation email upon publication.

The corresponding author will soon be receiving a typeset proof for review, to ensure errors have not been introduced during production. Please review the PDF proof of your manuscript carefully, as this is the last chance to correct any scientific or type-setting errors. Please note that major changes, or those which affect the scientific understanding of the work, will likely cause delays to the publication date of your manuscript. Note: Proofs for Front Matter articles (Editorial, Viewpoint, Symposium, Review, etc...) are generated on a different schedule and may not be made available as quickly.

Soon after your final files are uploaded, the early version of your manuscript will be published online unless you opted out of this process. The date of the early version will be your article's publication date. The final article will be published to the same URL, and all versions of the paper will be accessible to readers.

For Research Articles, you will receive an invoice from PLOS for your publication fee after your manuscript has reached the completed accept phase. If you receive an email requesting payment before acceptance or for any other service, this may be a phishing scheme. Learn how to identify phishing emails and protect your accounts at https://explore.plos.org/phishing.

Thank you again for supporting open-access publishing; we are looking forward to publishing your work in PLOS Neglected Tropical Diseases.

Best regards,

Shaden Kamhawi

co-Editor-in-Chief

PLOS Neglected Tropical Diseases

Paul Brindley

co-Editor-in-Chief

PLOS Neglected Tropical Diseases
